# The homeoprotein DLX4 controls inducible nitric oxide synthase-mediated angiogenesis in ovarian cancer

**DOI:** 10.1186/s12943-015-0368-3

**Published:** 2015-04-30

**Authors:** Bon Trinh, Song Yi Ko, Dhwani Haria, Nicolas Barengo, Honami Naora

**Affiliations:** Department of Molecular and Cellular Oncology, University of Texas MD Anderson Cancer Center, Houston, TX 77030 USA

**Keywords:** Angiogenesis, Ovarian cancer, Nitric oxide synthase, Homeobox gene

## Abstract

**Background:**

Homeobox genes encode transcription factors that control patterning of virtually all organ systems including the vasculature. Tumor angiogenesis is stimulated by several homeobox genes that are overexpressed in tumor cells, but the mechanisms of these genes are poorly understood. In this study, we investigated the mechanisms by which *DLX4,* a homeobox gene that is associated with increased tumor microvessel density, stimulates ovarian tumor angiogenesis.

**Methods:**

Expression of DLX4 and nitric oxide synthases was analyzed in publicly available transcriptional profiles of ovarian cancer clinical specimens. Levels of inducible nitric oxide synthase (iNOS) were evaluated by quantitative RT-PCR, flow cytometry and nitric oxide assays using ovarian cancer cell lines in which DLX4 was overexpressed or knocked down. Signal Transducer and Activator of Transcription 1 (STAT1) expression and activity were evaluated by luciferase reporter assays, immunofluorescence staining, Western blot and immunoprecipitation. Endothelial cell growth and tumor angiogenesis were evaluated in *in vitro* assays and xenograft models.

**Results:**

We identified that DLX4 induces expression of iNOS, an enzyme that stimulates angiogenesis by generating nitric oxide. Analysis of datasets of two independent patient cohorts revealed that high DLX4 expression in ovarian cancer is strongly associated with elevated expression of iNOS but not of other nitric oxide synthases. Studies using STAT1-expressing and STAT1-deficient cells revealed that DLX4 interacts with STAT1 and induces iNOS expression in part by stimulating STAT1 activity. Expression of DLX4 in ovarian cancer cells stimulated endothelial cell growth *in vitro* and increased microvessel density in xenograft models, and these stimulatory effects of DLX4 were abrogated when its induction of iNOS was inhibited.

**Conclusion:**

These findings indicate that DLX4 promotes ovarian tumor angiogenesis in part by stimulating iNOS expression.

**Electronic supplementary material:**

The online version of this article (doi:10.1186/s12943-015-0368-3) contains supplementary material, which is available to authorized users.

## Background

Angiogenesis is a tightly regulated process that is essential for normal organ development, tissue repair and regeneration, and tumor growth. Growth, maturation and migration of endothelial cells and vessel formation are controlled by environmental cues such as nitric oxide (NO) and hypoxia, and a network of growth factors, receptors and transcription factors [1-3]. Among the transcription factors that control angiogenesis, the most extensively studied include hypoxia-inducible factors (HIF), nuclear factor κB (NF-κB) and members of the Signal Transducer and Activator of Transcription (STAT) and E-twenty-six (ETS) families of transcription factors [3-6].

The homeobox gene super-family comprises more than 200 vertebrate genes and encodes transcription factors, termed homeoproteins, that are characterized by their conserved helix-turn-helix DNA-binding domain [7]. Homeoproteins are expressed in a temporal- and tissue- specific manner and play essential roles in controlling cell lineage-specification and tissue morphogenesis [7-9]. Increasing evidence indicates that angiogenesis is tightly regulated by specific sets of homeoproteins. HOXA9 promotes endothelial cell migration and tube formation by activating transcription of the gene encoding ephrin B4 [10]. HOXB5 promotes endothelial sprouting by inducing angiopoietin-2 expression [11]. In contrast, HOXA5, HOXD10 and GAX exert inhibitory effects on angiogenesis by various mechanisms such as inducing expression of thrombospondin-2 and p21WAF1/CIP1 [12-14].

Many homeobox genes have been found to be up- or down- regulated in a variety of tumors [8,9]. The mechanisms of most of these genes in tumor growth and progression are poorly understood as only few transcriptional targets have been identified. Notably, several homeoproteins that are aberrantly expressed in tumor cells have been found to control expression of pro-angiogenic growth factors. HOXB7 is overexpressed in more than 50% of breast cancers and activates the gene that encodes fibroblast growth factor-2 [15]. Conversely, loss of NKX3.1, which occurs in 80% of prostate cancers, increases expression of vascular endothelial growth factor (VEGF)-C [16]. High tumor microvessel density is strongly predictive of poor outcomes in patients with ovarian cancer [17,18]. We previously identified that DLX4, a homeoprotein that is absent from most normal adult tissues, is expressed in approximately 50% of ovarian cancers and is strongly associated with reduced survival [19]. Studies using xenograft models revealed that DLX4 expression in ovarian cancer cells increases tumor microvessel density, implicating a pro-angiogenic function for DLX4 [19]. Inducible nitric oxide synthase (iNOS) is an enzyme that promotes angiogenesis by generating NO [2,20] and its expression in ovarian cancers is strongly associated with poor outcomes [21,22]. In this study, we identified that DLX4 induces iNOS expression in part by stimulating STAT1 activity and promotes ovarian tumor angiogenesis by inducing iNOS expression. These findings raise the possibility that specific sets of homeoproteins promote tumor angiogenesis not only by directly regulating transcription of angiogenic growth factors, but also by modulating intracellular signaling and environmental cues.

## Results

### DLX4 induces iNOS expression

**Figure 1:**
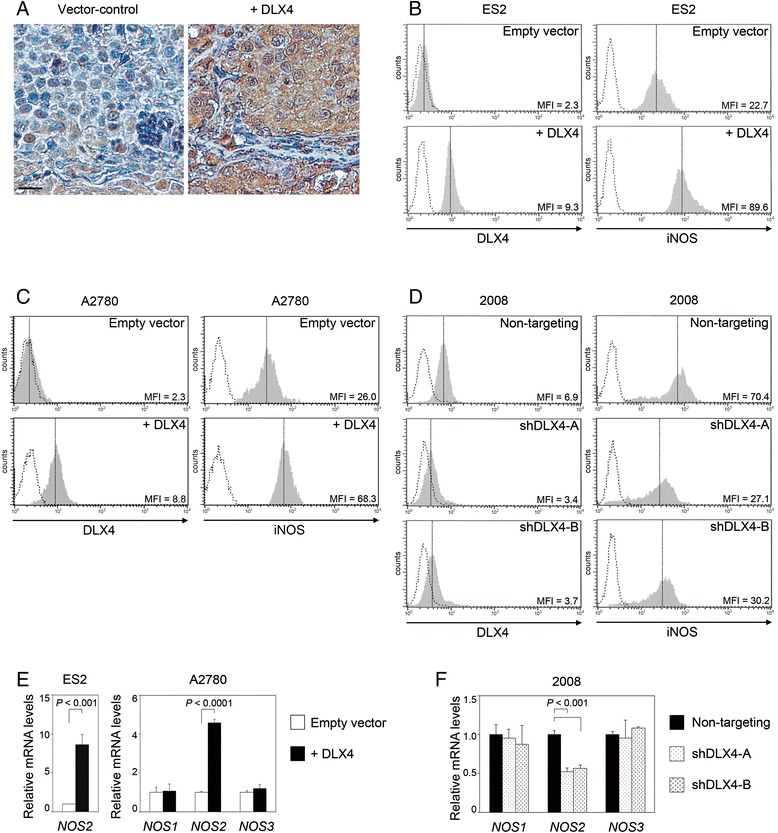
DLX4 induces iNOS expression. **(A)** Staining of iNOS in sections of peritoneal tumors of mice that were inoculated with vector-control and +DLX4 ES2 lines. Bar, 20 μm. **(B, C and D)** Flow cytometric analysis of intracellular staining of DLX4 and iNOS in transfected ovarian cancer cell lines. Mean fluorescence intensities (MFI) of staining are indicated. Shown are representative examples of DLX4 and iNOS staining in **(B)** vector-control and +DLX4 ES2 cells, **(C)** vector-control and +DLX4 A2780 cells and **(D)** 2008 cells transfected with non-targeting shRNA and shRNAs that targeted two different regions of *DLX4* (shDLX4-A, shDLX4-B). **(E)** qRT-PCR analysis of relative *NOS2* mRNA levels in ES2 cells and *NOS1, NOS2* and *NOS3* mRNA levels in A2780 cells. Levels of each mRNA in +DLX4 cells are expressed relative to the level in vector-control cells. **(F)** qRT-PCR analysis of relative *NOS1, NOS2* and *NOS3* mRNA levels in 2008 cells. Levels of each mRNA in *DLX4* shRNA-transfected cells are expressed relative to the level in non-targeting shRNA-transfected cells.

In previous studies, we identified that DLX4 increases tumor microvessel density in xenograft models of ovarian cancer, but the underlying mechanism of DLX4 was not clear [19]. Because iNOS is frequently expressed in ovarian cancers and stimulates tumor angiogenesis [2,20-22], we investigated the possibility that DLX4 stimulates iNOS expression. Stronger staining of iNOS was detected in xenografts derived from ES2 ovarian cancer cells that stably expressed DLX4 (+DLX4) than in xenografts derived from vector-control ES2 cells [Figure 1A]. Flow cytometric analysis of intracellular iNOS staining in cultured ES2 cells revealed that enforced expression of DLX4 induced a 4-fold increase in iNOS levels [Figure 1B]. Similarly, enforced expression of DLX4 in A2780 ovarian cancer cells induced iNOS levels [Figure 1C]. To confirm our findings, we evaluated iNOS levels when endogenous DLX4 was knocked down by two different *DLX4* shRNAs (shDLX4-A, shDLX4-B). Levels of iNOS were decreased when endogenous DLX4 was knocked down in 2008 cells [Figure 1D]. Furthermore, iNOS levels were decreased when DLX4 was knocked down in three additional ovarian cancer cell lines (OVCAR8, OVCA429 and TOV112D) [Additional file 1: Figures S1 A,B and C]. Changes in iNOS expression were confirmed by quantitative reverse transcription PCR (qRT-PCR) analysis of *NOS2* mRNA levels. *NOS2* mRNA levels significantly increased when DLX4 was overexpressed (*P* < 0.001) [Figure 1E], and decreased when DLX4 was knocked down (*P* < 0.001) [Figure 1F]. We also assayed mRNA levels of *NOS1* (encoding neuronal nitric oxide synthase nNOS) and *NOS3* (encoding endothelial nitric oxide synthase eNOS). *NOS1* and *NOS3* mRNA levels were detected in A2780 and 2008 cells, but were almost undetectable in ES2 cells and other ovarian cancer cell lines that we tested. In contrast to *NOS2*, mRNA levels of *NOS1* and *NOS3* did not significantly change when DLX4 was overexpressed in A2780 cells or when DLX4 was knocked down in 2008 cells [Figure 1E and F].

### Elevated expression of DLX4 is associated with increased iNOS expression in ovarian cancer clinical specimens

**Figure 2:**
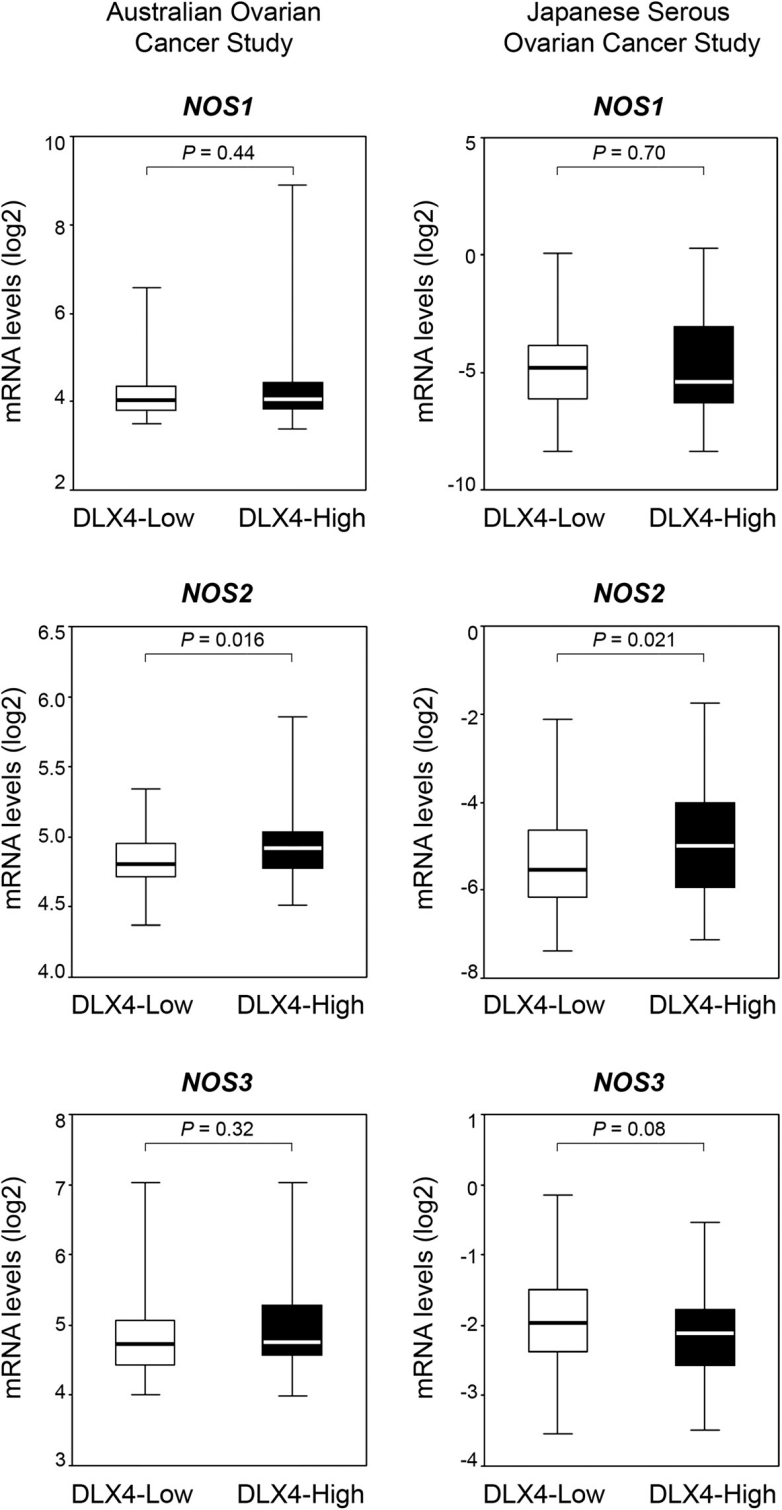
High expression of DLX4 is associated with increased iNOS expression in clinical specimens of ovarian cancer. Cases from the Australian Ovarian Cancer Group Study [23] (GSE9891, n = 285) were stratified according to *DLX4* expression in tumors, where *DLX4* mRNA levels were defined as High (≥ upper quartile) and Low (≤ lower quartile). Significance of differences in *NOS1, NOS2* and *NOS3* mRNA levels (log2 scale) between upper and lower quartile sub-groups was evaluated by Mann–Whitney *U*-test. The same analysis was performed for cases from the Japanese Serous Ovarian Cancer Group Study [24] (GSE32062, n = 260).

To evaluate whether iNOS expression is elevated in ovarian cancers that highly express DLX4, we analyzed published, publicly available transcriptional profiles of clinical specimens from the Australian Ovarian Cancer Group Study [23]. Cases from this dataset (n =285) were stratified into quartile sub-groups according to the levels of *DLX4* transcripts in tumors. Levels of *NOS2* transcripts were significantly higher in *DLX4*-High tumors (upper quartile sub-group) than in *DLX4-*Low tumors (lower quartile sub-group) (*P* = 0.016) [Figure 2]. Levels of *NOS2* transcripts were also significantly higher in *DLX4*-High tumors than in *DLX4-*Low tumors (*P* = 0.021) in an independent patient cohort (n = 260 cases) from the Japanese Serous Ovarian Cancer Group Study [24]. In contrast to *NOS2*, no significant differences were found in expression levels of *NOS1* and *NOS3* between *DLX4*-High and *DLX4*-Low cases in the two cohorts [Figure 2]. These observations are consistent with our findings that DLX4 induces expression of *NOS2* but does not alter expression of *NOS1* or *NOS3* in ovarian cancer cells [Figure 1E and F].

### DLX4 induces iNOS expression in a STAT1-dependent manner

**Figure 3:**
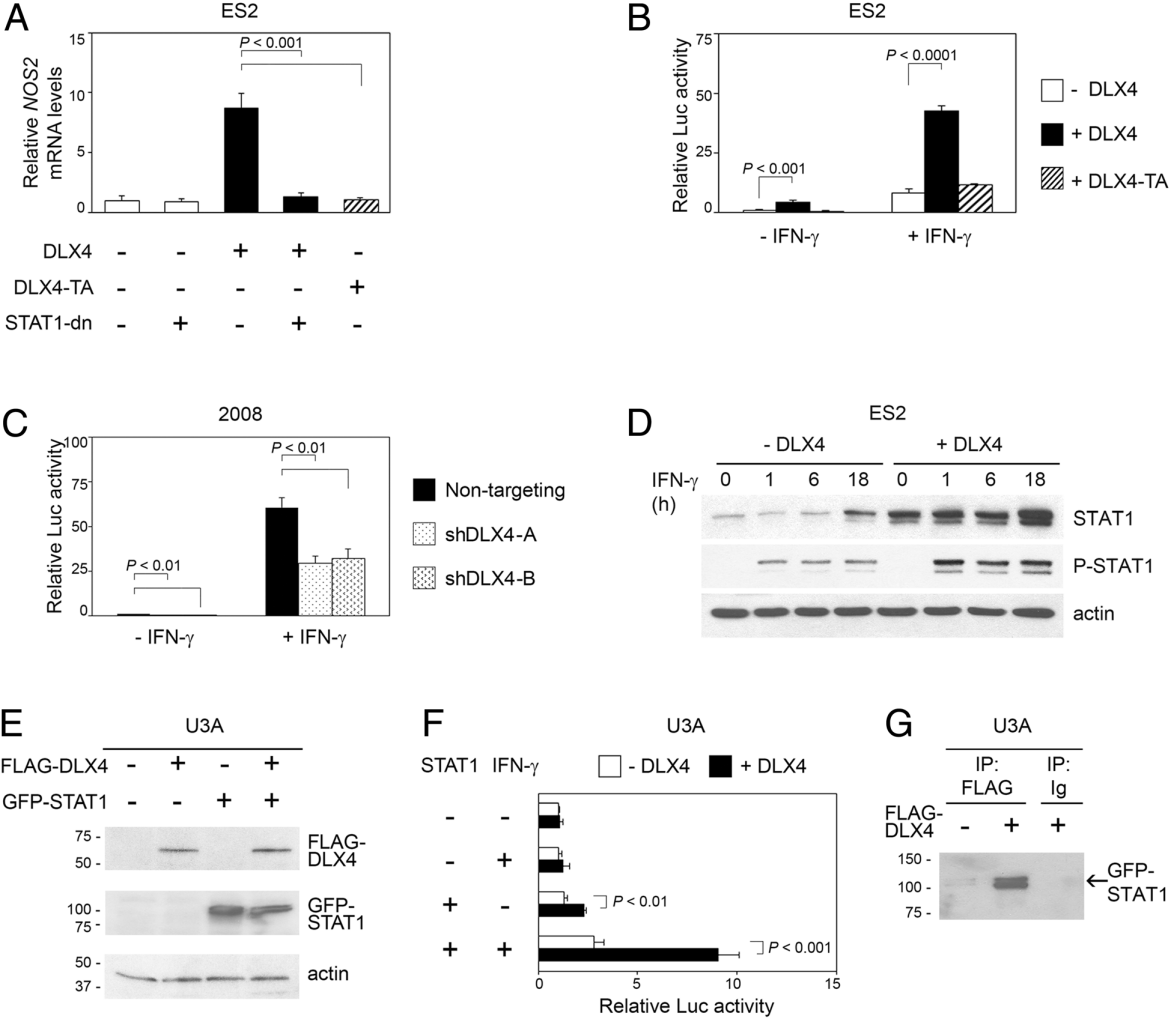
DLX4 stimulates STAT1 activity and induces iNOS expression in a STAT1-dependent manner. **(A)** qRT-PCR analysis of relative *NOS2* mRNA levels in vector-control ES2 cells and in ES2 cells that expressed wild-type DLX4 or mutant DLX4 (DLX4-TA) with or without dominant-negative STAT1 (STAT1-dn). **(B)** Vector-control ES2 cells and ES2 cells that expressed wild-type or mutant DLX4 were transfected with a firefly luciferase reporter construct driven by GAS elements (GAS-LUC), stimulated without or with IFN-γ (10 ng/mL) for 16 h and then assayed for luciferase activity. **(C)** Activity of the GAS-LUC reporter construct was assayed in 2008 cells that expressed non-targeting and *DLX4* shRNAs as described in **(B)**. **(D)** Western blot analysis of levels of total STAT1 and phosphorylated STAT1 in vector-control and +DLX4 ES2 cells that were stimulated with IFN-γ (10 ng/mL) for 0, 1, 6 and 18 h. **(E)** Lysates of U3A cell lines that lacked or stably expressed GFP-STAT1 fusion protein and/or FLAG-tagged DLX4 fused to GFP were assayed by Western blot using Abs to STAT1 and DLX4. **(F)** Activity of the GAS-LUC reporter construct in STAT1-deficient U3A cells and in U3A cells reconstituted with STAT1 that lacked or expressed DLX4. Transfected cells were stimulated with or without IFN-γ (10 ng/mL) for 16 h and then assayed for luciferase activity. **(G)** FLAG Ab was used to pull down FLAG-tagged DLX4 in U3A cells that were stimulated with IFN-γ (10 ng/mL) for 1 h. Immunoprecipitates were analyzed by Western blot using Ab to STAT1. Pulldown using control Ig was included as a negative control. Shown in **B**, **C** and **F** are relative firefly luciferase activities in three independent experiments.

DLX4 has been reported to regulate expression of several genes by modulating activity of other transcription factors such as Smad4 and Sp1 [25]. STAT1 is a potent transcriptional activator of *NOS2* [26,27]. Induction of *NOS2* mRNA levels by DLX4 in ES2 cells was abrogated by a dominant-negative STAT1 Y701F mutant [28] (STAT1-dn) (*P* < 0.001) [Figure 3A]. We also evaluated the effect of a mutant form of DLX4 (DLX4-TA) that lacks the C-terminal region and is unable to translocate into the nucleus [25,29]. This DLX4 mutant did not induce *NOS2* mRNA levels [Figure 3A]. Together, these findings indicated that DLX4 induces iNOS expression in a STAT1-dependent manner and raised the possibility that DLX4 might stimulate STAT1 activity. To evaluate the effect of DLX4 on STAT1 activity, we assayed activity of a luciferase reporter construct driven by STAT1-binding, interferon (IFN) Gamma-Activated Sites (GAS) elements (GAS-LUC). Enforced expression of wild-type DLX4 in ES2 cells significantly induced GAS-LUC reporter activity (*P* < 0.0001) [Figure 3B]. In contrast, GAS-LUC reporter activity was not induced by the DLX4-TA mutant [Figure 3B]. To confirm our findings, we evaluated GAS-LUC reporter activity when endogenous DLX4 was knocked down. GAS-LUC reporter activity was inhibited when DLX4 was knocked down in 2008 cells (*P* < 0.01) [Figure 3C] and also in OVCAR8 and TOV112D cells (*P* < 0.01) [Additional file 1: Figure S2].

### DLX4 interacts with STAT1 and stimulates STAT1 expression and activity

To further investigate the stimulatory effect of DLX4 on STAT1, we evaluated its effects on STAT1 phosphorylation and expression. Following IFN-γ stimulation, higher levels of phosphorylated STAT1 were detected in +DLX4 ES2 cells than in vector-control ES2 cells [Figure 3D]. Consistent with this observation, higher levels of STAT1 were detected in the nucleus of +DLX4 ES2 cells [Additional file 1: Figure S3]. The increases in STAT1 phosphorylation and nuclear localization in +DLX4 cells corresponded to an increase in the expression level of STAT1 [Figure 3D]. Because transcription of the *STAT1* gene is controlled in part by a GAS element [30], we evaluated the effect of DLX4 on STAT1 activity independently of its induction of STAT1 expression. To accomplish this, we used the STAT1-deficient fibrosarcoma cell line U3A [31] as a model and reconstituted STAT1 expression in this cell line under the control of the constitutive CMV promoter [Figure 3E]. DLX4 was stably expressed in both STAT1-deficient and STAT1-reconstituted U3A cells [Figure 3E]. DLX4 significantly induced GAS-LUC reporter activity in U3A cells that stably expressed STAT1 (*P* < 0.001) [Figure 3F]. This finding indicated that DLX4 stimulates STAT1 activity *per se.* Immunoprecipitation (IP) assays revealed that DLX4 interacts with STAT1 [Figure 3G]. DLX4 might therefore induce iNOS expression by interacting with and stimulating STAT1 activity.

### DLX4 stimulates VEGF-A production and endothelial cell growth *in vitro* by inducing iNOS

**Figure 4:**
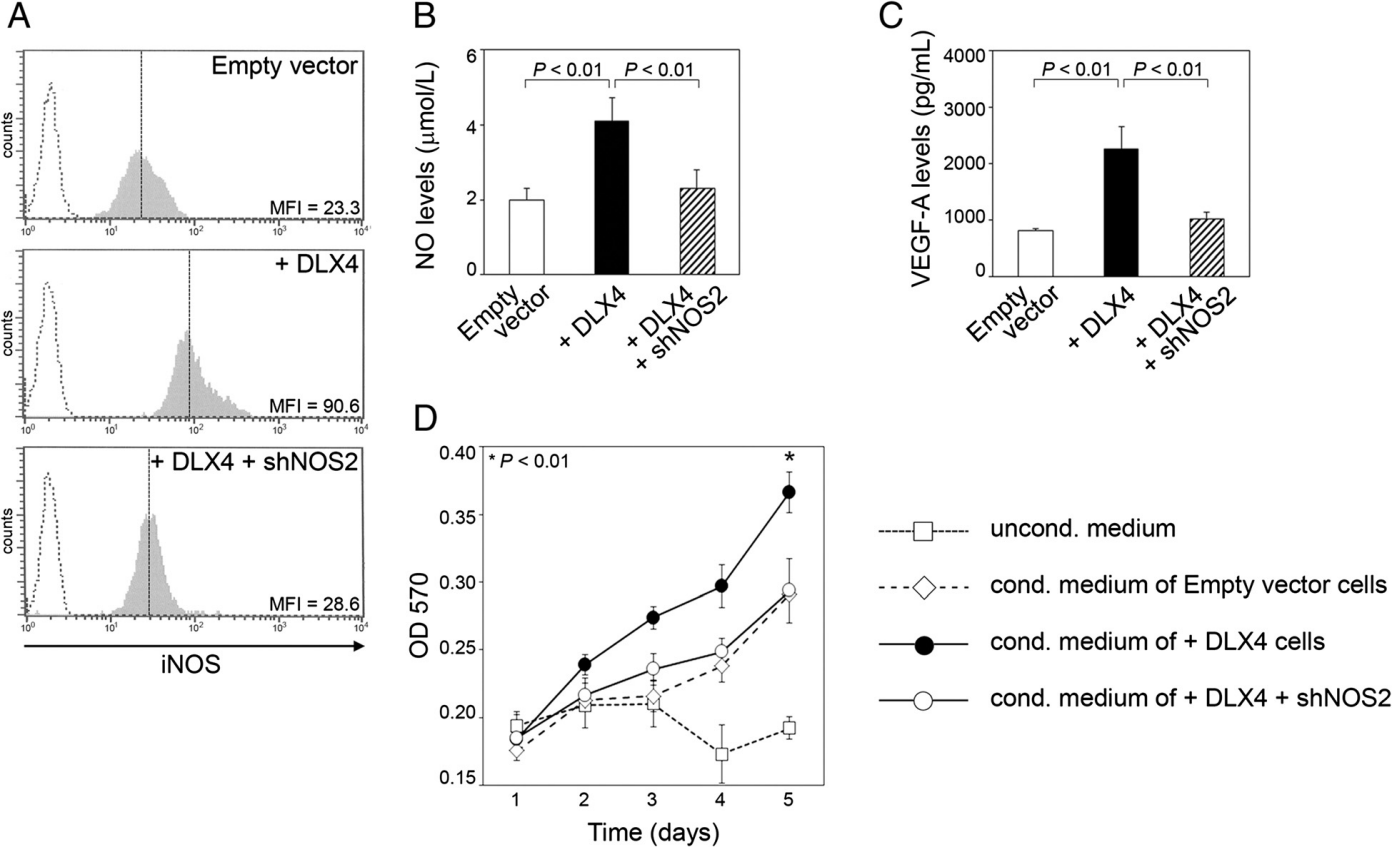
DLX4 stimulates NO levels, VEGF-A production and endothelial cell growth *in vitro* by inducing iNOS. **(A)** Flow cytometric analysis of iNOS levels in vector-control and +DLX4 ES2 cells, and in +DLX4 ES2 cells that stably expressed *NOS2* shRNA (+DLX4 + shNOS2). **(B)** NO levels were assayed by using Griess reagent in culture medium that was conditioned by equivalent numbers of cells of each ES2 line. **(C)** Levels of VEGF-A were assayed by ELISA in tumor-conditioned medium. **(D)** Growth of endothelial cells cultured in tumor-conditioned medium was evaluated by MTT assay. Shown in **B**, **C** and **D** are average results of three independent experiments.

A major mechanism by which iNOS promotes angiogenesis is by generating NO that stimulates VEGF-A expression [32,33]. In subsequent experiments, we sought to determine whether DLX4 stimulates NO and VEGF-A production in tumor cells by inducing iNOS. We evaluated whether the stimulatory effects of DLX4 are abrogated when its induction of iNOS is inhibited. To accomplish this, we stably knocked down the level of iNOS in +DLX4 ES2 cells almost to the level seen in vector-control ES2 cells [Figure 4A]. Levels of NO and VEGF-A were assayed in cell culture medium that was conditioned by equivalent numbers of cells of each ES2 line. DLX4 induced levels of NO and VEGF-A (*P* < 0.01), and this induction was abrogated when iNOS was knocked down (i.e. +DLX4 + shNOS2) [Figure 4B and C]. Endothelial cell growth was more highly stimulated by medium that was conditioned by +DLX4 cells than by medium conditioned by vector-control cells (*P* < 0.01) [Figure 4D]. In contrast, medium conditioned by +DLX4 + shNOS2 cells was only as effective as medium conditioned by vector-control cells in stimulating endothelial cell growth [Figure 4D]. These findings indicate that the stimulatory effects of DLX4 on VEGF-A production and endothelial cell growth are substantially mediated through its induction of iNOS.

### Stimulatory effects of DLX4 on ovarian tumor angiogenesis and ascites formation are mediated through its induction of iNOS *in vivo*

**Figure 5:**
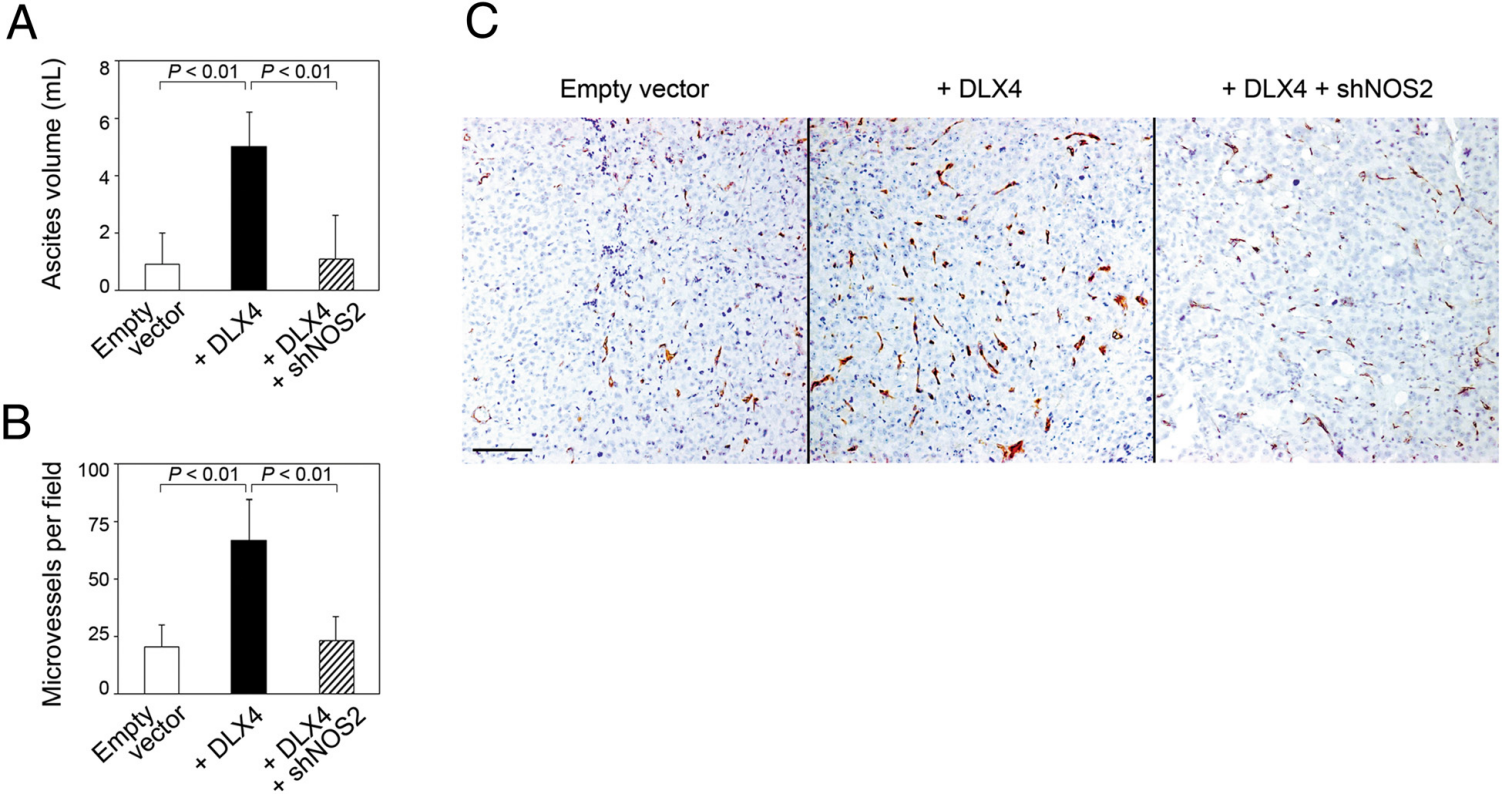
DLX4 stimulates tumor angiogenesis and ascites formation in i.p. xenograft models of ovarian cancer by inducing iNOS. Female nude mice (n = 5 per group) were inoculated i.p. with equivalent numbers of cells (1 x 10^6^) of vector-control, +DLX4 and +DLX4 + shNOS2 ES2 lines and sacrificed at 20 days thereafter. **(A)** Volume of ascites. **(B)** Average numbers of microvessels were calculated in omental tumors by scoring five random 100x microscopic fields of CD34-stained tissue sections of each mouse. **(C)** Representative examples of CD34 staining in omental tumors. Bar, 100 μm.

To confirm our *in vitro* findings, we evaluated whether DLX4 stimulates ovarian tumor angiogenesis via its induction of iNOS by generating mouse i.p. xenograft models from vector-control and +DLX4 ES2 cells and from +DLX4 ES2 cells in which iNOS was knocked down. Enforced expression of DLX4 induced ascites formation, and knockdown of iNOS in +DLX4 models reduced ascites almost to the level seen in vector-control models [Figure 5A]. Consistent with our previous studies [19], DLX4 increased tumor microvessel density [Figure 5B and C]. Knockdown of iNOS in +DLX4 xenograft models reduced microvessel density to a level comparable to that seen in vector-control models [Figure 5B and C]. Together, our findings indicate that the stimulatory effect of DLX4 on ovarian tumor angiogenesis is substantially mediated through its induction of iNOS.

## Discussion

Several homeoproteins that are expressed in endothelial cells play important roles in transcriptional programs that control normal endothelial cell growth, migration and tube formation [10-14]. Increasing evidence indicates that aberrant expression of other sets of homeoproteins in tumor cells increases tumor angiogenesis (reviewed in Ref. [9]), but their mechanisms are poorly understood. In this study, we identified that DLX4, a homeoprotein that is overexpressed in ovarian cancer, stimulates ovarian tumor angiogenesis by inducing iNOS expression. To our knowledge, our study is the first to identify a role for a homeoprotein in controlling NO generation. NO is generated from L-arginine, NADPH and oxygen by NO synthases of which there are three isoforms [2,20]. nNOS and eNOS are constitutively expressed, predominantly in neuronal cells and endothelial cells, respectively, and are also expressed in several types of tumors including a subset of ovarian cancers [2,20,34]. iNOS produces substantially more NO than the other NO synthase isoforms [2]. We found that high DLX4 expression is associated with increased expression of iNOS but not of nNOS or eNOS in clinical specimens of ovarian cancer, and that DLX4 induces expression of iNOS but not of nNOS or eNOS in ovarian tumor cells. A principal mechanism by which NO promotes angiogenesis is by increasing VEGF-A production [32,33]. Our findings that the stimulatory effects of DLX4 on VEGF-A production and tumor angiogenesis are abrogated when iNOS is inhibited support the conclusion that these stimulatory effects of DLX4 are primarily mediated via its induction of iNOS. In addition to its potent angiogenic property, VEGF-A causes ascites formation in ovarian cancer [35]. DLX4 expression in ovarian tumor cells induced ascites formation in mice and this induction was abrogated when iNOS was inhibited. Agents that neutralize VEGF-A or inhibit VEGF receptor tyrosine kinase activity have been extensively evaluated in clinical trials of ovarian cancer patients and a limitation of these agents is acquired resistance [36]. Inhibition of iNOS might be an effective approach to inhibit tumor angiogenesis and also ascites formation in ovarian cancer.

The findings of the present study indicate that DLX4 interacts with STAT1 and induces iNOS expression at least in part by stimulating STAT1 activity. Although dominant-negative STAT1 abrogated the ability of DLX4 to induce iNOS expression, we cannot eliminate the possibility that DLX4 might also stimulate activity of other transcription factors that activate *NOS2* transcription such as NF-κB and HIF-1α [27,37]. To our knowledge, our study is the first to identify an interaction between a homeoprotein and a member of the STAT family. Although the precise mechanism by which DLX4 stimulates STAT1 activity remains to be determined, it is possible that DLX4 stabilizes STAT1-containing transcriptional complexes. Whereas inflammatory cytokines induce *NOS2* transcription, transforming growth factor-β (TGF-β) decreases *NOS2* mRNA stability and translation and increases degradation of iNOS protein [38]. We previously identified that DLX4 blocks TGF-β/Smad signaling [25]. It is therefore possible that DLX4 might also increase levels of iNOS by blocking TGF-β-mediated *NOS2* mRNA destabilization and iNOS protein degradation. Whereas iNOS induces VEGF-A production, VEGF-A stimulation reciprocally increases expression of iNOS [39]. DLX4 might additionally, through its induction of iNOS, enhance the iNOS-VEGF-A regulatory loop in tumors.

Whereas NO has a pro-tumorigenic effect by stimulating angiogenesis, continuous exposure to NO at high concentrations exerts genotoxic effects [20]. Metabolites of NO can cause DNA damage and lead to cell death [20]. NO has been reported to induce expression of the catalytic subunit of DNA-dependent protein kinase (DNA-PK), an essential component of the non-homologous end-joining (NHEJ) DNA repair machinery [40]. This NO-mediated induction of DNA-PK has been found to protect cells from the deleterious effects of NO and other DNA damaging agents [40]. We recently identified that DLX4 stimulates DNA-PK activity and NHEJ-mediated DNA repair by interacting with Ku70 and Ku80 that bind to DNA double-strand break ends [29]. This activity of DLX4 protected tumor cells against DNA damaging chemotherapeutic agents such as doxorubicin and etoposide [29]. It is possible that DLX4 might similarly protect tumor cells against the genotoxic effects of high NO levels by stimulating repair of DNA damage caused by NO metabolites. In addition, several studies have identified that STAT1 confers resistance to DNA damage (reviewed in Ref. [41]). DLX4 might also confer resistance to NO-induced DNA damage by stimulating STAT1 activity.

Because relatively few transcriptional targets of homeoproteins have been identified, it is unclear how the tumor-promoting properties of these factors are related to their normal developmental functions. The *DLX4* gene was originally identified in a screen of a placental cDNA library [42] but its normal function is poorly understood. Preeclampsia is characterized by reduced placental blood flow and elevated maternal blood pressure, and is a major cause of fetal and maternal morbidity and mortality [43]. Substantial evidence indicates that disruption of NO bioavailability contributes to the pathophysiology of preeclampsia and that expression of iNOS is reduced in preeclamptic placentas [43-45]. Interestingly, it has also been reported that DLX4 expression is down-regulated in preeclamptic placentas [46]. Our findings that DLX4 induces iNOS expression raise the intriguing possibility that down-regulation of DLX4 in the placenta might promote endothelial dysfunction in preeclampsia by causing a reduction in iNOS levels.

## Conclusion

In summary, the present study supports increasing evidence of the significance of homeoproteins in the regulation of angiogenesis. Whereas prior studies have shown that homeoproteins control expression of angiogenic growth factors or receptors [10,11,13,15,16], our findings indicate that homeoproteins can also promote angiogenesis by modulating intracellular signaling and environmental cues. Because homeoproteins are transcription factors that share a conserved DNA-binding domain, it could be difficult to therapeutically target these proteins with high specificity. A peptide that disrupts interactions of specific HOX proteins with PBX co-factors has been reported to inhibit tumor cell growth [47]. It might therefore be possible to inhibit the tumor-promoting functions of homeoproteins such as DLX4 by disrupting their interactions with binding partners.

## Methods

### Plasmids

The pIRES-EGFP2 plasmid encoding FLAG-tagged DLX4 has been previously described [25]. The *DLX4* cDNA was subcloned into the pRetroQ-AcGFP retroviral vector (Clontech). Other plasmids were as follows: pGFP-V-RS plasmids containing non-targeting and *DLX4* shRNAs (OriGene Technologies), pGIPZ plasmids containing *NOS2* shRNAs (GE Healthcare), eGFP-STAT1 (provided by Alan Perantoni, National Cancer Institute, Frederick, MD; Addgene plasmid 12301) [48], pRc/CMV-Flag STAT1α Y701F [28] (provided by James Darnell, Rockefeller University, New York, NY; Addgene plasmid 8702). A firefly luciferase reporter construct driven by tandem GAS elements was purchased from SABiosciences.

### Antibodies (Abs) and other reagents

Sources of Abs were as follows: DLX4, iNOS, CD34 (Abcam), STAT1, phosphorylated STAT1 (Y701) (Cell Signaling Technology), FLAG, actin (Sigma-Aldrich), secondary Abs (BD Biosciences, Invitrogen). Recombinant IFN-γ was purchased from R&D Systems.

### Cell culture and transfection

Culture media were purchased from Invitrogen and were supplemented with penicillin-streptomycin and 10% fetal bovine serum. Vector-control and DLX4-overexpressing ES2 cell lines have been previously described [19] and were cultured in McCoys’ 5A medium. The parental 2008 cell line was provided by Zahid Siddik (MD Anderson Cancer Center, Houston, TX) and cultured in RPMI 1640 medium. Parental A2780, OVCAR8 and OVCA429 cell lines were provided by Gordon Mills (MD Anderson Cancer Center) and cultured in RPMI 1640 medium (A2780, OVCAR8) and Dulbecco’s Modified Eagle’s Medium (DMEM) (OVCA429). The parental TOV112D cell line was provided by Ju-Seog Lee (MD Anderson Cancer Center) and cultured in a 1:1 mixture of MCDB 105 medium and Medium 199. The U3A cell line [31] was provided by George Stark (Cleveland Clinic Lerner Research Institute, Cleveland, OH) and cultured in DMEM. Immortalized mouse ovarian endothelial cells [49] were provided by Isaiah Fidler (MD Anderson Cancer Center) and cultured in DMEM medium. Ampho-293 cells were provided by Douglas Boyd (MD Anderson Cancer Center) and cultured in DMEM. Ampho-293 cells were transfected with pRetroQ-AcGFP retroviral constructs by using Lipofectamine®2000 reagent (Invitrogen). At 48 h thereafter, viral supernatants were harvested and used to infect U3A and A2780 cells. In other experiments, cultured cells were directly transfected with plasmids by using Lipofectamine®2000 reagent or FuGENE®6 reagent (Promega). Cells were selected by addition of G418 (400 μg/mL) or puromycin (0.5 μg/mL).

### NO assay

Cells were cultured overnight in medium that contained no FBS or Phenol Red. Culture supernatants were thereafter depleted of proteins that were >10 kD in size by centrifugation at 14,000 × *g* for 10 mins through Amicon Ultra 0.5 mL centrifugal filters (Millipore). NO levels in supernatants were assayed by using Griess reagent (Total NO and Nitrate/Nitrite Parameter Assay Kit, R&D Systems) following manufacturer’s instructions. Three independent experiments were performed for each assay.

### Conditioned medium and cell proliferation assays

Equivalent numbers of tumor cells (1.5×10^6^) were seeded in 10 cm dishes and cultured in medium containing 2% FBS for 2 d. Tumor-conditioned medium was assayed for VEGF-A levels by ELISA (R&D Systems) and added to endothelial cells. Endothelial cell growth was measured by using the 3-(4,5-dimethylthiazolyl-2)-2,5-diphenyltetrazolium bromide (MTT) assay (Roche). Three independent experiments were performed for each assay.

### qRT-PCR

*NOS1, NOS2* and *NOS3* mRNA levels were analyzed by using primers and SYBR®Green qPCR Master Mix that were purchased from SABiosciences. *RPL32* transcript levels were used as controls for normalization and were detected by using the following primers: forward: 5′-ACAAAGCACATGCTGCCCAGTG-3′, reverse: 5′- TTCCACGATGGCTTTGCGGTTC-3′.

### Immunofluorescence staining of cultured cells

For analysis of staining by flow cytometry, cells were fixed in 1% paraformaldehyde (20 min at 4°C) and permeabilized in 0.1% saponin (15 min at room temperature). Thereafter, cells were incubated with Abs to DLX4 (1:20) or to iNOS (1:100) for 30 min at 4°C, washed and incubated with peridinin-chlorophyll-protein complex-conjugated secondary Ab. Staining was detected by flow cytometry (FACS Calibur, BD Biosciences). For analysis of staining by fluorescence microscopy, cells were plated in chamber slides, fixed in 4% paraformaldehyde (20 min at 4°C) and permeabilized in 0.1% Triton-X100 (15 min at room temperature). Thereafter, cells were incubated with STAT1 Ab (1:100) for 30 min at 4°C, washed and incubated with Alexa Fluor 594-conjugated secondary Ab. Cells were stained with diamidino-2-phenylindole (DAPI) (Sigma-Aldrich) and then viewed using an Eclipse 80i fluorescence microscope (Nikon).

### IP and immunoblotting

Cell extracts were prepared by lysing cells in M-PER buffer (Pierce Biotechnology). For IP, 2 mg of cell extract was pre-cleared with protein G agarose and incubated with FLAG Ab or with control Ig using lysis buffer (20 mM Tris–HCl pH 8.0, 100 mM NaCl, 1% Nonidet P-40, 10% glycerol, 2 mM EDTA). Cell extracts and immunoprecipitates were subjected to SDS-PAGE and immunoblotting using polyvinylidene fluoride membrane (GE Healthcare).

### Reporter assays

Cells were co-transfected with plasmids containing *DLX4* cDNA or shRNAs, firefly luciferase reporter plasmid and Renilla luciferase reporter plasmid to normalize transfection efficiency as previously described [25]. Where indicated, recombinant IFN-γ (10 ng/mL) was added to cells at 24 h after transfection. At 16 h thereafter, luciferase activities were assayed by using the Dual-luciferase reporter assay kit (Promega). Three independent experiments were performed for each assay.

### Mouse i.p. xenografts

All animal studies were approved by the University of Texas MD Anderson Cancer Center Institutional Animal Care and Use Committee. Four-week-old female nude mice were purchased from Charles River and inoculated i.p. with 1 × 10^6^ cells of ES2 lines (n = 5 mice per group). At 3 weeks thereafter, mice were euthanized by CO_2_ asphyxiation. Volume of ascites was measured. Sections of formalin-fixed, paraffin-embedded tissues were stained with Abs to iNOS and CD34 and staining detected by streptavidin-biotin-peroxidase and 3,3′-diaminobenzidine (Dako). Microvessels were counted in 5 independent and random 100x microscopic fields in stained tumor tissue sections of each mouse.

### Bioinformatic analysis

Published gene expression data from Australian Ovarian Cancer Group Study [23] (GSE9891, n = 285) and from the Japanese Serous Ovarian Cancer Group Study [24] (GSE32062, n = 260) were downloaded from the National Center for Biotechnology Information Gene Expression Omnibus (GEO) database (http://www.ncbi.nlm.nih.gov/geo). Patients were stratified according to expression of *DLX4* in tumors, where *DLX4* transcript levels were defined as High (≥ upper quartile) and Low (≤ lower quartile).

### Statistical analysis

STATISTICA6 software (StatSoft Inc.) was used for statistical analysis. Statistical significance of data of *in vitro* and *in vivo* assays was assessed by unpaired two-tailed Student’s *t-*test. Significance of differences in gene expression between groups of patients was assessed by Mann–Whitney *U*-test. *P* values of < 0.05 were considered significant.

## Additional file

Additional file 1: Figure S1. Related to Figure 1. Knockdown of DLX4 decreases iNOS levels in ovarian tumor cells. Flow cytometric analysis of intracellular staining of DLX4 and iNOS in **(A)** OVCAR8, **(B)** OVCA429 and **(C)** TOV112D cells, where cells of these lines were transfected with non-targeting shRNA and shRNAs that targeted two different regions of *DLX4* (shDLX4-A, shDLX4-B). Shown are representative examples of staining and mean fluorescence intensities (MFI). Solid grey histograms represent staining with Abs to DLX4 and to iNOS. Dotted histograms represent staining with isotype control. **Figure S2.** Related to Figure 3. DLX4 stimulates STAT1-driven promoter activity. OVCAR8 and TOV112D cells that expressed non-targeting and *DLX4* shRNAs were transfected with the GAS-LUC reporter construct, stimulated without or with IFN-γ (10 ng/mL) for 16 h and then assayed for luciferase activity. Shown are relative firefly luciferase activities in three independent experiments. **Figure S3.** Related to Figure 3. Effect of DLX4 on STAT1 localization in ovarian tumor cells**.** Immunofluorescence staining of STAT1 (shown in red) in vector-control and +DLX4 ES2 cells that were stimulated with IFN-γ (10 ng/mL) for 1 h or left unstimulated. Cells were also stained with DAP1 (shown in blue) to visualize nuclei. Bar, 20 μm.

## Abbreviations

Ab: antibody
DNA-PK: DNA-dependent protein kinase
eNOS: endothelial nitric oxide synthase
GAS: IFN Gamma-Activated Sites
HIF: hypoxia-inducible factor
IFN: interferon
iNOS: inducible nitric oxide synthase
IP: immunoprecipitation
NF-κB: nuclear factor κB
NHEJ: non-homologous end-joining
nNOS: neuronal nitric oxide synthase
NO: nitric oxide
qRT-PCR: quantitative reverse transcription PCR
shRNAs: short hairpin RNAs
STAT: Signal Transducer and Activator of Transcription
TGF-β: transforming growth factor-β
VEGF: vascular endothelial growth factor
