# Supplementary material for: The homeoprotein DLX4 controls inducible nitric oxide synthase-mediated angiogenesis in ovarian cancer
Source: Mol Cancer. 2015 Apr 30;14:97. doi: 10.1186/s12943-015-0368-3 (PMC4427985; doi:10.1186/s12943-015-0368-3)

SUPPLEMENTAL FIGURE LEGENDS

Supplemental Figure S1. Knockdown of DLX4 decreases iNOS levels in ovarian tumor cells. Flow cytometric analysis of intracellular staining of DLX4 and iNOS in **(A)** OVCAR8, **(B)** OVCA429 and **(C)** TOV112D cells, where cells of these lines were transfected with non-targeting shRNA and shRNAs that targeted two different regions of *DLX4* (shDLX4-A, shDLX4-B). Mean fluorescence intensities (MFI) of staining are indicated.

Supplemental Figure S2. DLX4 stimulates STAT1-driven promoter activity. OVCAR8 and TOV112D cells that expressed non-targeting and *DLX4* shRNAs were transfected with the GAS-LUC reporter construct, stimulated without or with IFN-γ (10 ng/mL) for 16 h and then assayed for luciferase activity. Shown are relative firefly luciferase activities in three independent experiments.

Supplemental Figure S3. Effect of DLX4 on STAT1 localization in ovarian tumor cells. Immunofluorescence staining of STAT1 (shown in red) in vector-control and +DLX4 ES2 cells that were stimulated with IFN-γ (10 ng/mL) for 1 h or left unstimulated. Cells were also stained with DAP1 (shown in blue) to visualize nuclei. Bar, 20 μm.

Supplemental Figure S1

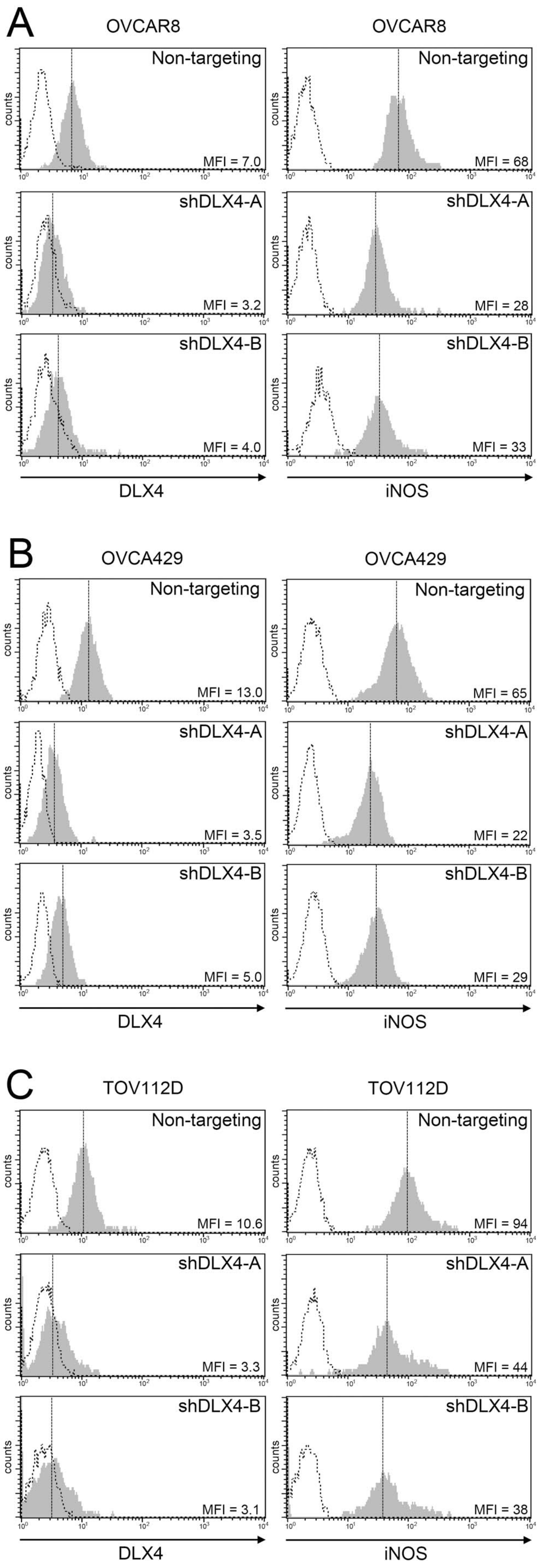

Supplemental Figure S2

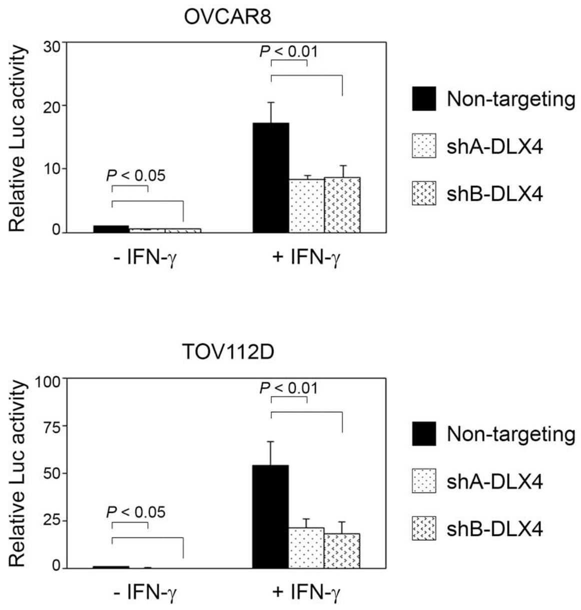

Supplemental Figure S3

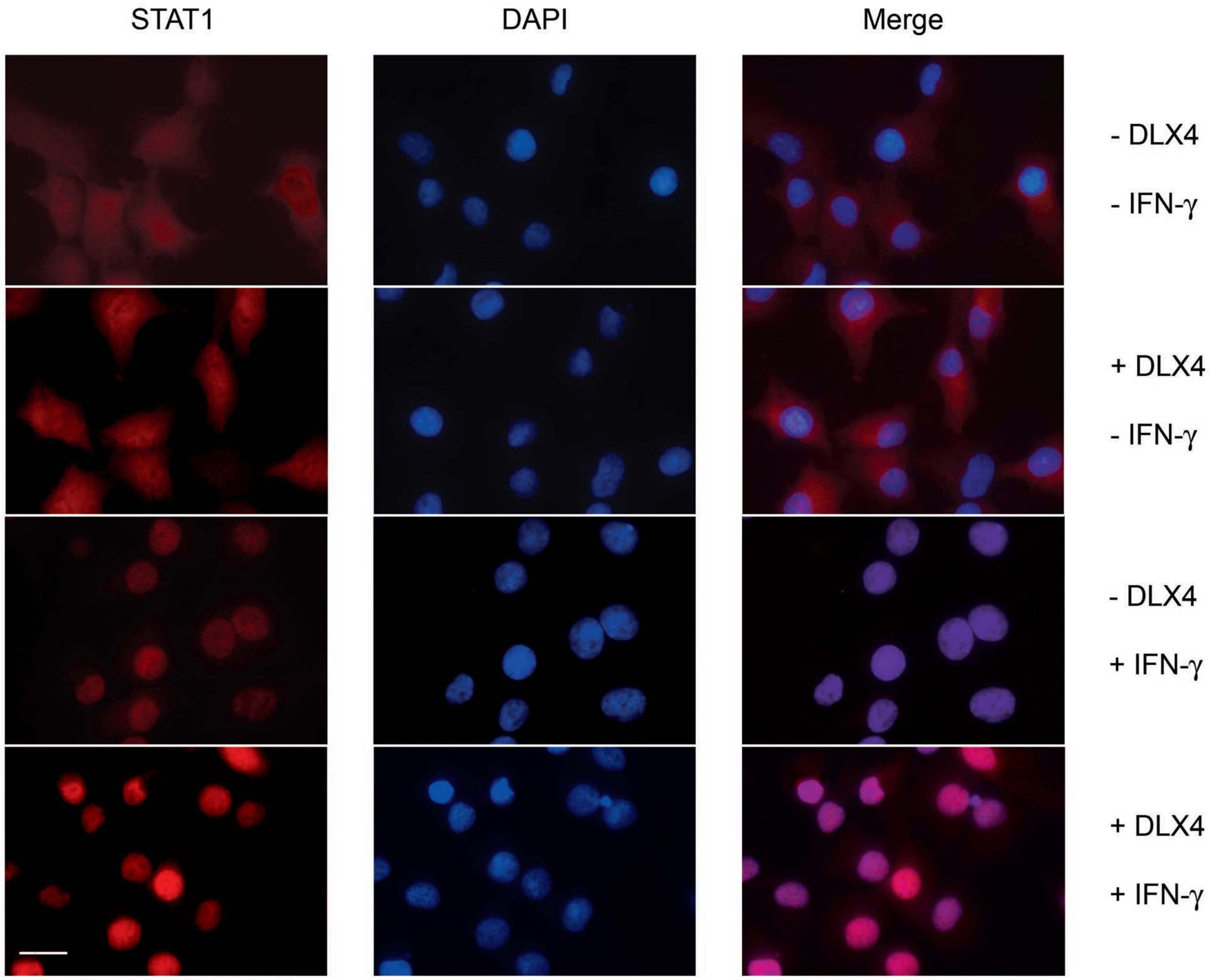

Supplement: Additional file 1: Figure S1. — Related to Figure 1. Knockdown of DLX4 decreases iNOS levels in ovarian tumor cells. Flow cytometric analysis of intracellular staining of DLX4 and iNOS in (A) OVCAR8, (B) OVCA429 and (C) TOV112D cells, where cells of these lines were transfected with non-targeting shRNA and shRNAs that targeted two different regions of DLX4 (shDLX4-A, shDLX4-B). Shown are representative examples of staining and mean fluorescence intensities (MFI). Solid grey histograms represent staining with Abs to DLX4 and to iNOS. Dotted histograms represent staining with isotype control. Figure S2. Related to Figure 3. DLX4 stimulates STAT1-driven promoter activity. OVCAR8 and TOV112D cells that expressed non-targeting and DLX4 shRNAs were transfected with the GAS-LUC reporter construct, stimulated without or with IFN-γ (10 ng/mL) for 16 h and then assayed for luciferase activity. Shown are relative firefly luciferase activities in three independent experiments. Figure S3. Related to Figure 3. Effect of DLX4 on STAT1 localization in ovarian tumor cells. Immunofluorescence staining of STAT1 (shown in red) in vector-control and +DLX4 ES2 cells that were stimulated with IFN-γ (10 ng/mL) for 1 h or left unstimulated. Cells were also stained with DAP1 (shown in blue) to visualize nuclei. Bar, 20 μm. [file 12943_2015_368_MOESM1_ESM.pdf]
